# Is Aging a Disease?

**DOI:** 10.1111/jocd.70535

**Published:** 2025-11-09

**Authors:** Oskar Brugrand

**Affiliations:** ^1^ Department of Dermatology South Infirmary Victoria University Hospital (SIVUH) Cork Ireland

In clinic, I have noticed two distinct kinds of patients. One sits with quiet acceptance: “Doctor, my wrinkles tell my story.” The other, equally thoughtful, confides: “I don't want the world to know my age.” Both seek care, yet their goals, and the social forces shaping them, differ profoundly.

Cosmetic dermatology has shifted from niche to routine practice [1]. Laser treatments, neuromodulators, fillers, and resurfacing now appear alongside acne therapy and skin cancer checks. For some, these procedures restore function, lifting eyelids that obstruct vision, improving post‐traumatic scars, or softening stigmatizing changes from disease. For others, they serve to ‘stay competitive’ in workplaces and social settings where youth is prized. The boundary between treatment and enhancement is often blurred.

The demand for anti‐aging care reflects more than personal preference; it mirrors society's discomfort with visible aging [2, 3]. The skin, our most public organ, becomes a billboard for age. Patients often describe feeling invisible or devalued once fine lines or pigmentation emerge [4, 5]. Even the term ‘age spots’ can carry a sting. This is not merely about beauty; it is the lived reality of ageism [4, 5]. When patients seek treatment to avoid discrimination, their decisions are shaped as much by cultural pressure as by personal desire [2].

For clinicians, this duality generates ambivalence. Dermatologists can affirm individuality by supporting a patient's goals, or risk reducing them to a homogenized, youthful ideal. Here lies an ethical tension. By providing cosmetic procedures, do we uphold patient autonomy, or inadvertently reinforce ideals that harm them? Unlike treating psoriasis or melanoma, cosmetic work does not restore a prior ‘normal’; it redefines normal itself [1]. Dermatologists can both challenge and perpetuate ageist norms [1, 5].

This tension is compounded by the subtle influence of *perception drift*. With repeated exposure to anti‐aging requests and before‐and‐after images, clinicians may unconsciously recalibrate what ‘healthy’ or ‘normal’ skin should look like. Over time, the threshold for what counts as a treatable defect narrows, aligning professional judgment with cultural ideals of youth. This drift risks depersonalizing patients, measuring them less by their own stories and more by standardized aesthetic templates, a phenomenon recognized as *perception drift* [6].

Some clinicians prioritize autonomy: if a safe, effective treatment is requested with informed consent, it is ethically permissible [1]. Others question whether fulfilling such requests perpetuates harmful standards [3]. Between these positions lies a middle ground, one that recognizes the influence of social forces [2, 4], addresses them openly, and supports shared decision‐making.

A practical approach is to integrate skin health and aesthetics in consultations [1]. Discussing sun protection, photoaging prevention, and topical therapies alongside procedural options signals that healthy skin is valued at every age [5]. Presenting non‐intervention as a legitimate choice helps destigmatize visible aging [2, 4]. The most satisfying outcomes often emerge when decisions are collaborative, values‐based, and free of judgment, whether the plan involves a laser session, a topical cream, or embracing natural aging.

Can dermatology ever be neutral in the face of ageism? Perhaps not [4, 5]. But by acknowledging our dual role as healers and participants in the beauty economy, we can act deliberately to minimize harm. Cosmetic interventions are not inherently unethical; context matters [1]. How they are offered, discussed, and justified determines whether they enhance well‐being or reinforce exclusionary ideals.

As populations age worldwide [5], this dilemma will become more pressing. The goal is not to choose between promoting healthy skin aging and addressing aesthetic preferences, but to integrate them, affirming that beauty, like health, exists across the lifespan [2]. The challenge is to help patients look the way they wish without framing aging itself as a disease [1, 3].

## Ethics Statement

The author has nothing to report.

## Consent

The author has nothing to report.

## Conflicts of Interest

The author declares no conflicts of interest.

## Data Availability

Data sharing not applicable to this article as no datasets were generated or analysed during the current study.

## References

[1] A. Misitzis , R. Gupta , J. Maghfour , et al., “The Ethics of Cosmetic Dermatology,” Clinics in Dermatology 38 (2020): 569–574.33280805

[2] M. M. Gullette , Aged by Culture (University of Chicago Press, 2004).

[3] D. Callahan , “The Scourge of Anti‐Aging Medicine,” Journals of Gerontology. Series A, Biological Sciences and Medical Sciences 59 (2004): B573–B579. 15215267 10.1093/gerona/59.6.b573

[4] T. D. Nelson , “Ageism: Prejudice Against Our Feared Future Self,” Journal of Social Issues 61 (2005): 207–221.

[5] World Health Organization , Global Report on Ageism (WHO, 2021).

[6] H. H. Sinno , S. C. Wilson , N. D. Brownstone , and M. R. Lee , “The Ethics of Cosmetic Overtreatment: The Phenomenon of Perception Drift,” Journal of Cutaneous Medicine and Surgery 27, no. 3 (2023): 288–293.37067257

